# Supplementary figures and images for: SNAP25 disease mutations change the energy landscape for synaptic exocytosis due to aberrant SNARE interactions
Source: eLife. 2024 Feb 27;12:RP88619. doi: 10.7554/eLife.88619 (PMC10911398; doi:10.7554/eLife.88619)

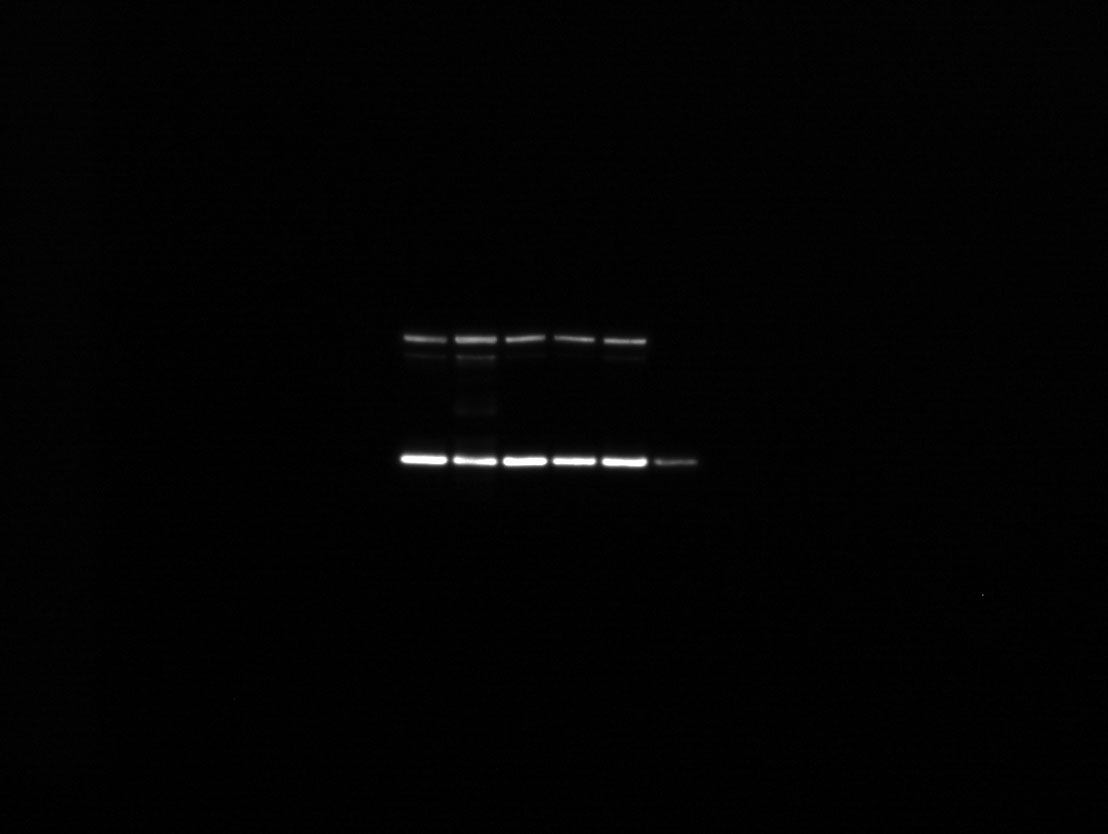

Supplement: Figure 2—source data 2. [file elife-88619-fig2-data2.zip › SNAP25_30s exposure.jpg]

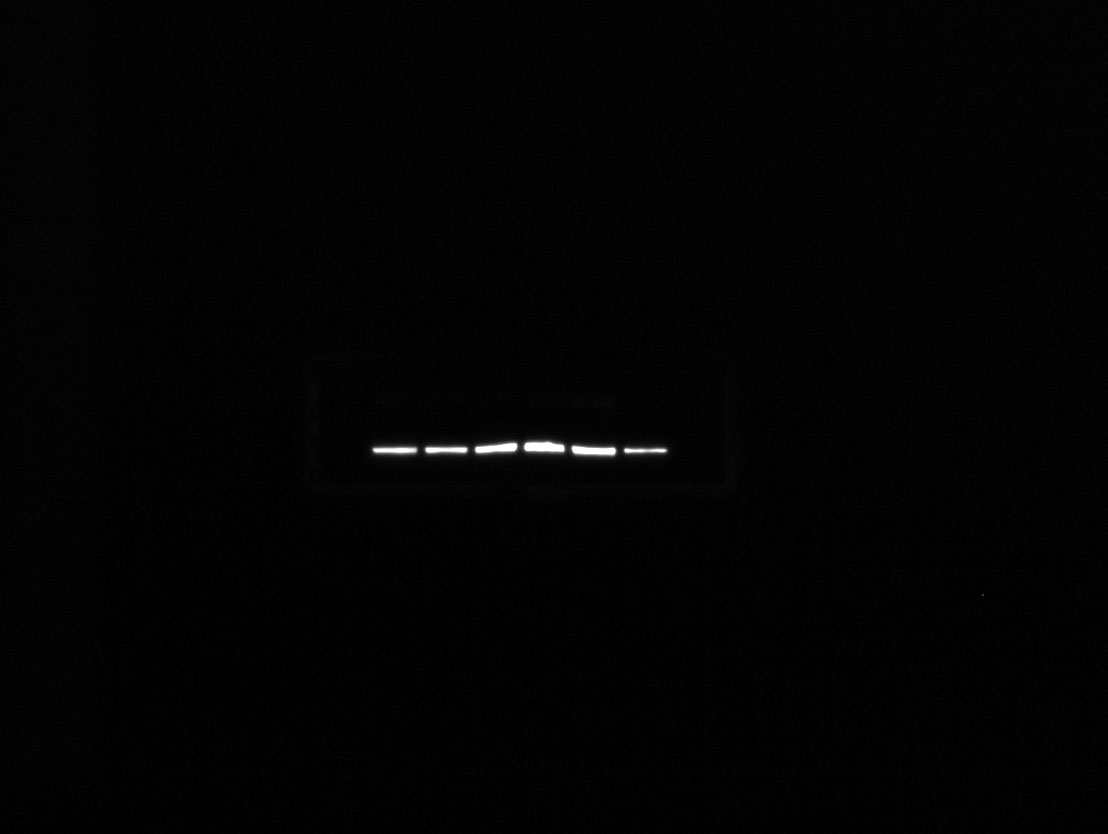

Supplement: Figure 2—source data 2. [file elife-88619-fig2-data2.zip › VCP_20s exposure.jpg]

VCP exposure time: 20s

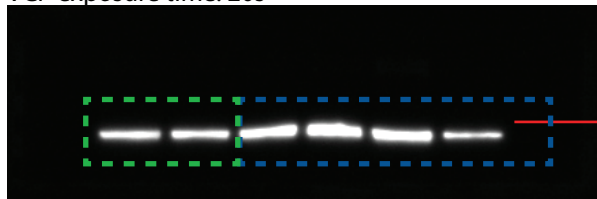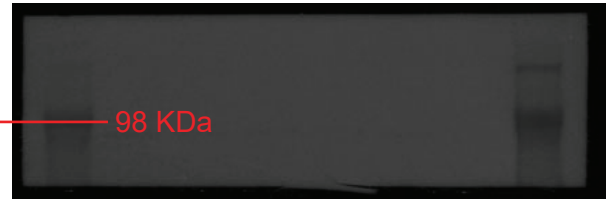

SNAP25 exposure time: 30s

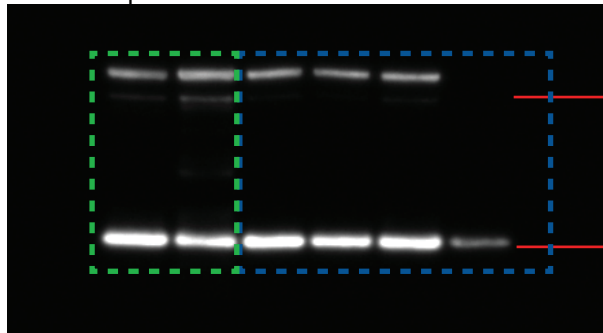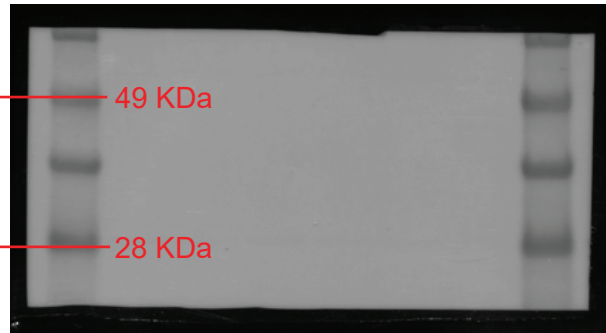

**Figure 2**

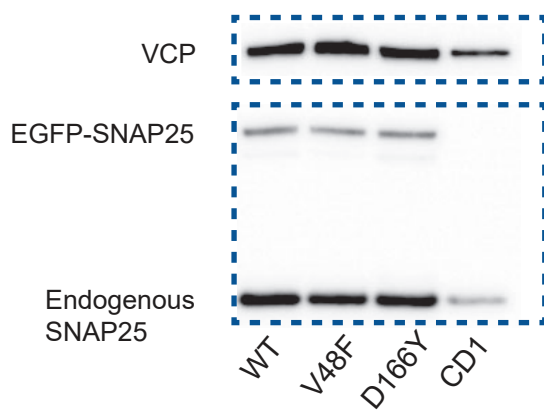

**Figure 10**

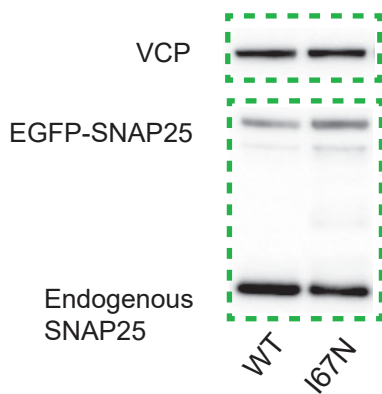

Supplement: Figure 2—source data 3. [file elife-88619-fig2-data3.zip › Raw images of WB membranes presented in figures 2 +10.pdf]

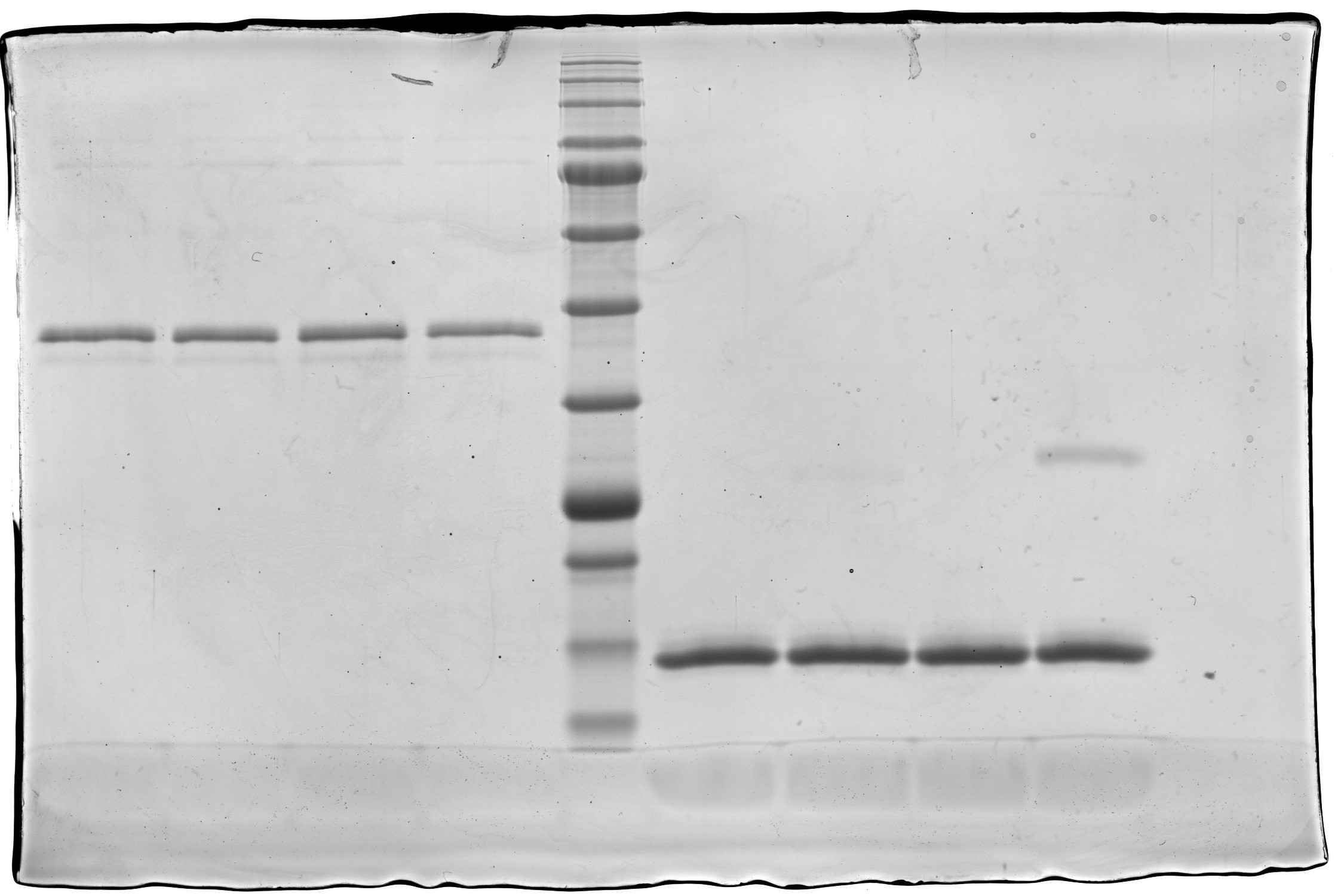

Supplement: Figure 9—figure supplement 1—source data 1. [file elife-88619-fig9-figsupp1-data1.zip › Figure 9 - figure Supplement 1 - Source data 1/Gel1.jpg]

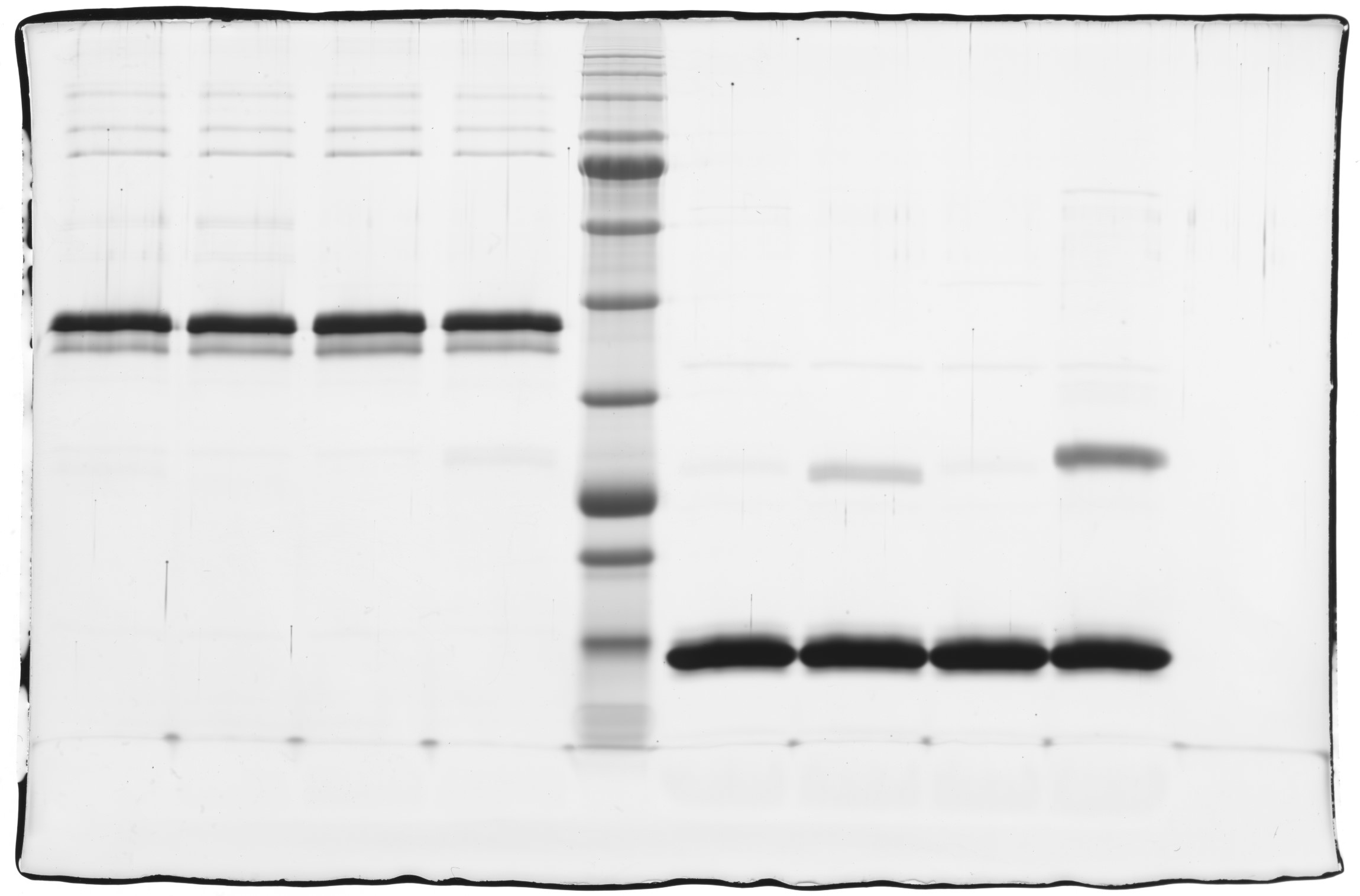

Supplement: Figure 9—figure supplement 1—source data 1. [file elife-88619-fig9-figsupp1-data1.zip › Figure 9 - figure Supplement 1 - Source data 1/Gel2.jpg]

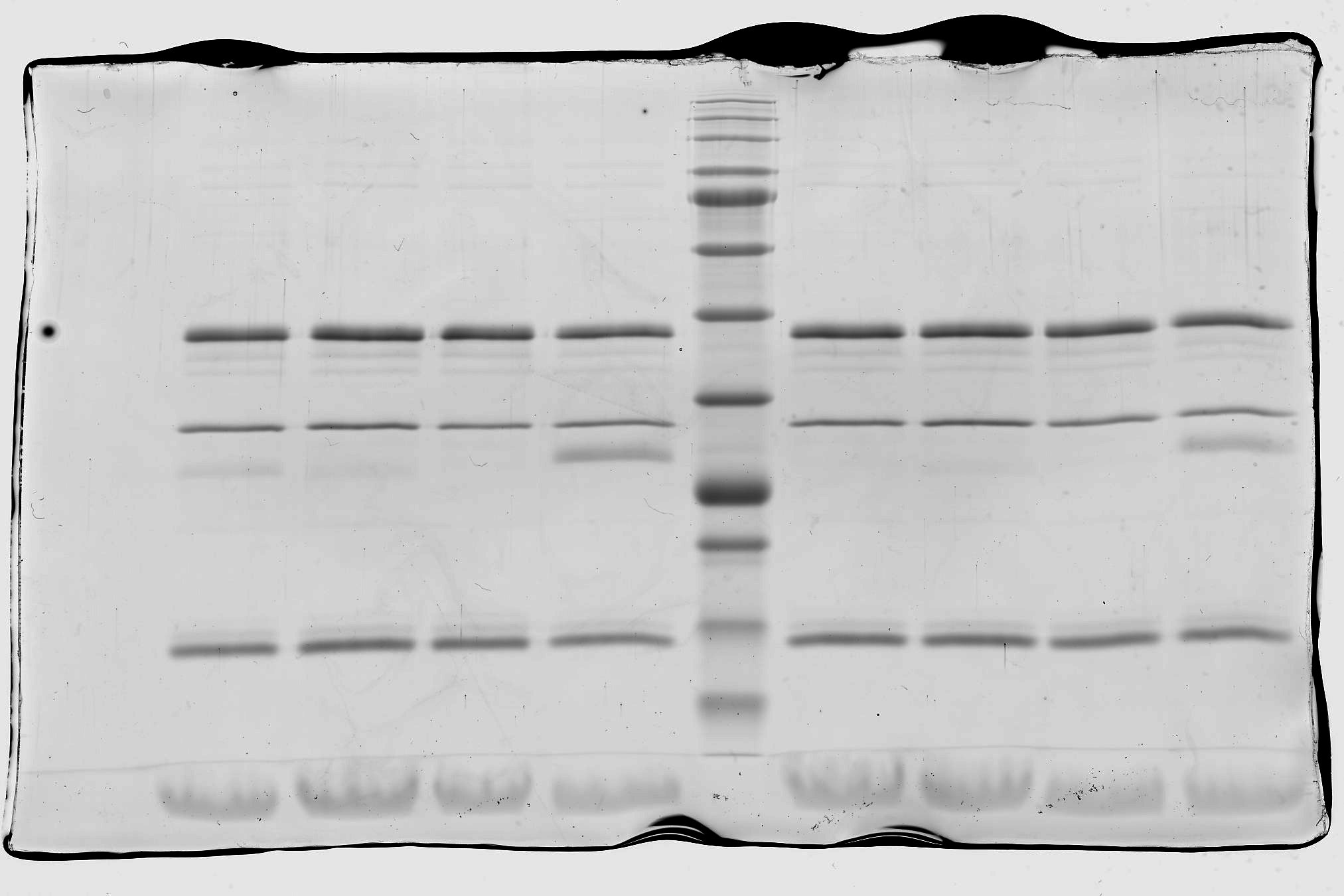

Supplement: Figure 9—figure supplement 1—source data 1. [file elife-88619-fig9-figsupp1-data1.zip › Figure 9 - figure Supplement 1 - Source data 1/Gel3.jpg]

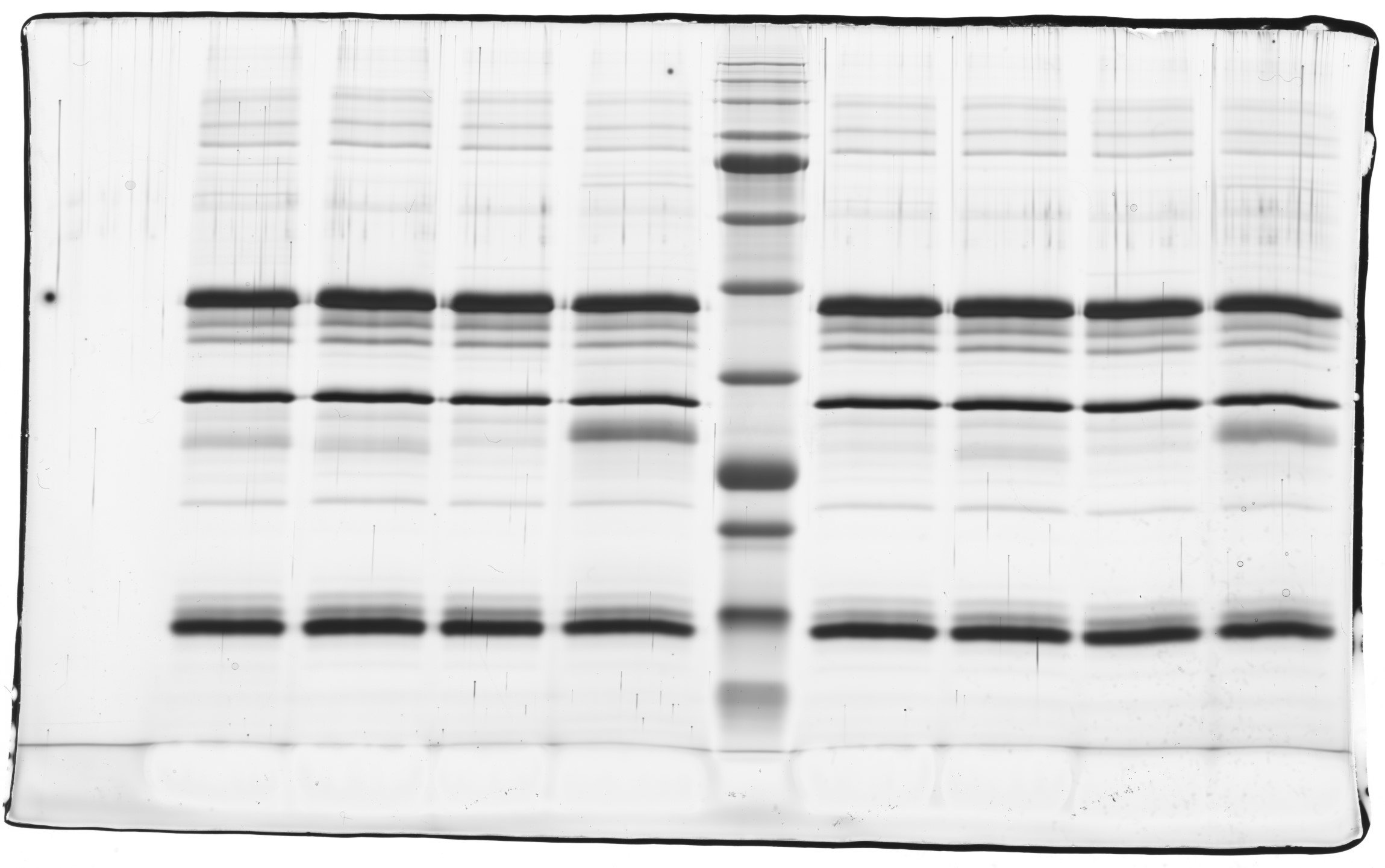

Supplement: Figure 9—figure supplement 1—source data 1. [file elife-88619-fig9-figsupp1-data1.zip › Figure 9 - figure Supplement 1 - Source data 1/Gel4.jpg]

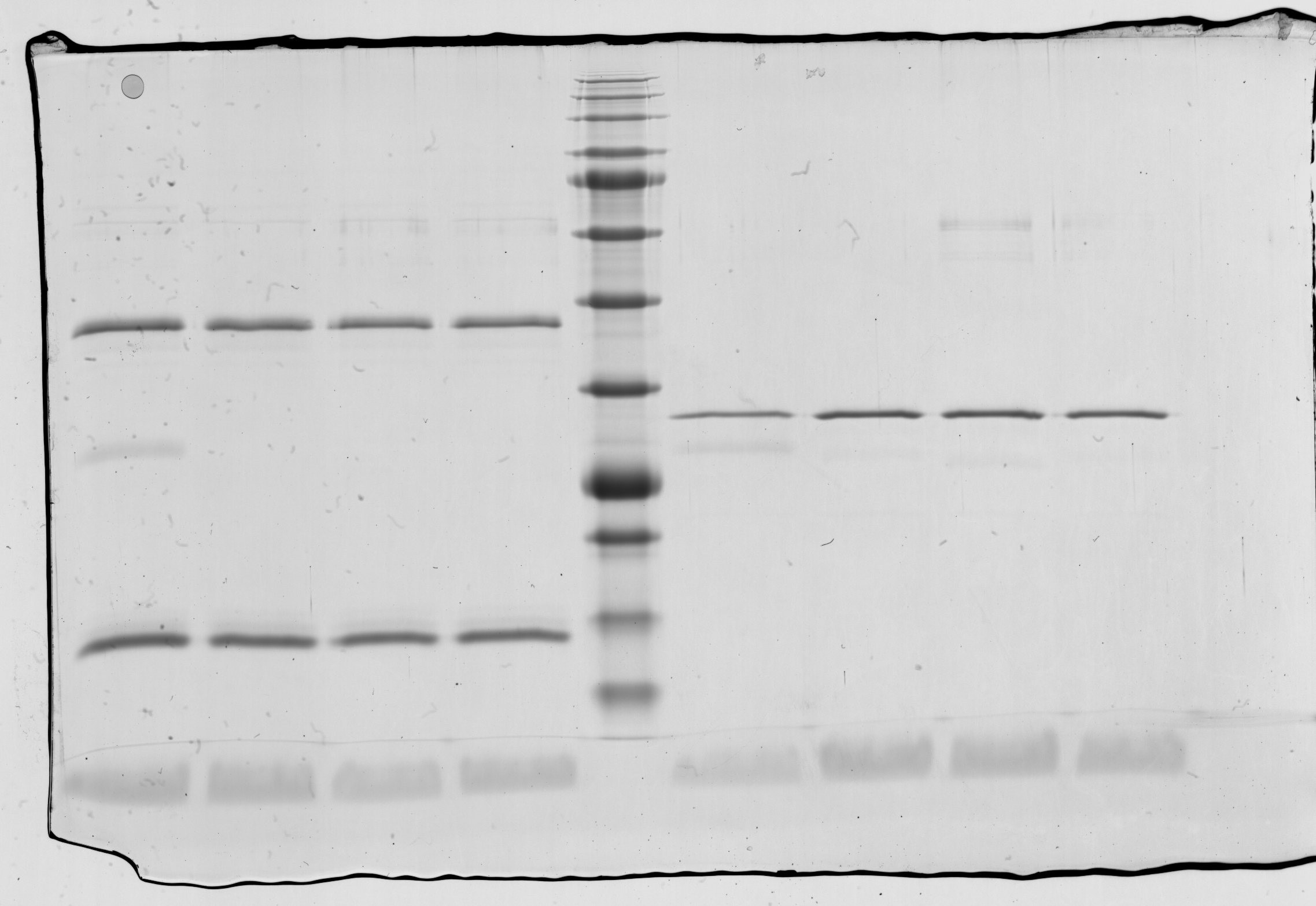

Supplement: Figure 9—figure supplement 1—source data 1. [file elife-88619-fig9-figsupp1-data1.zip › Figure 9 - figure Supplement 1 - Source data 1/Gel5.jpg]

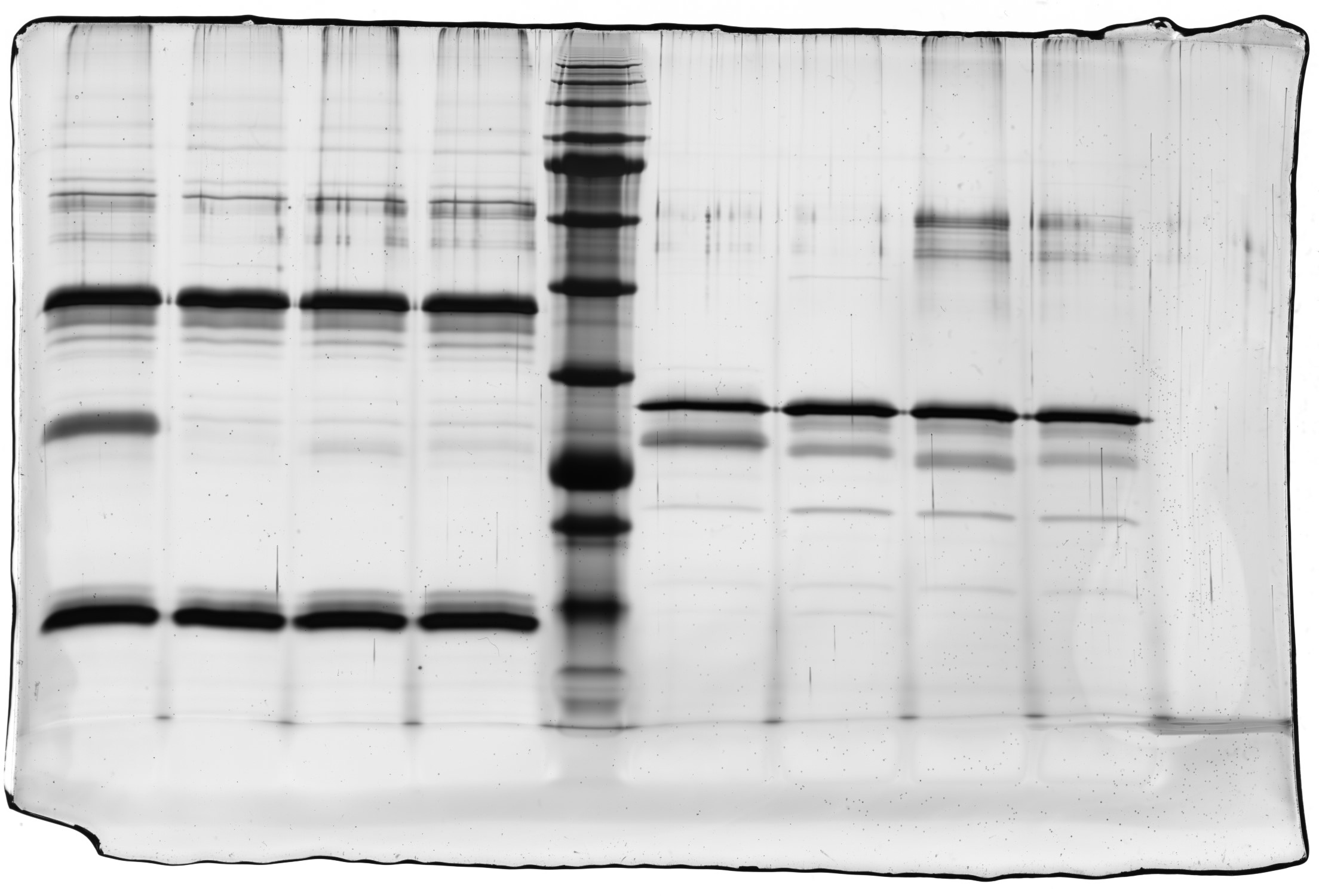

Supplement: Figure 9—figure supplement 1—source data 1. [file elife-88619-fig9-figsupp1-data1.zip › Figure 9 - figure Supplement 1 - Source data 1/Gel6.jpg]
