# Supplementary material for: SNAP25 disease mutations change the energy landscape for synaptic exocytosis due to aberrant SNARE interactions
Source: eLife. 2024 Feb 27;12:RP88619. doi: 10.7554/eLife.88619 (PMC10911398; doi:10.7554/eLife.88619)
Supplement: Figure 9—figure supplement 1—source data 2. [file elife-88619-fig9-figsupp1-data2.zip › Figure 9 - Figure Supplement 1 - Source Data 1.pdf]

Figure 9 – Supplement 1

Raw data

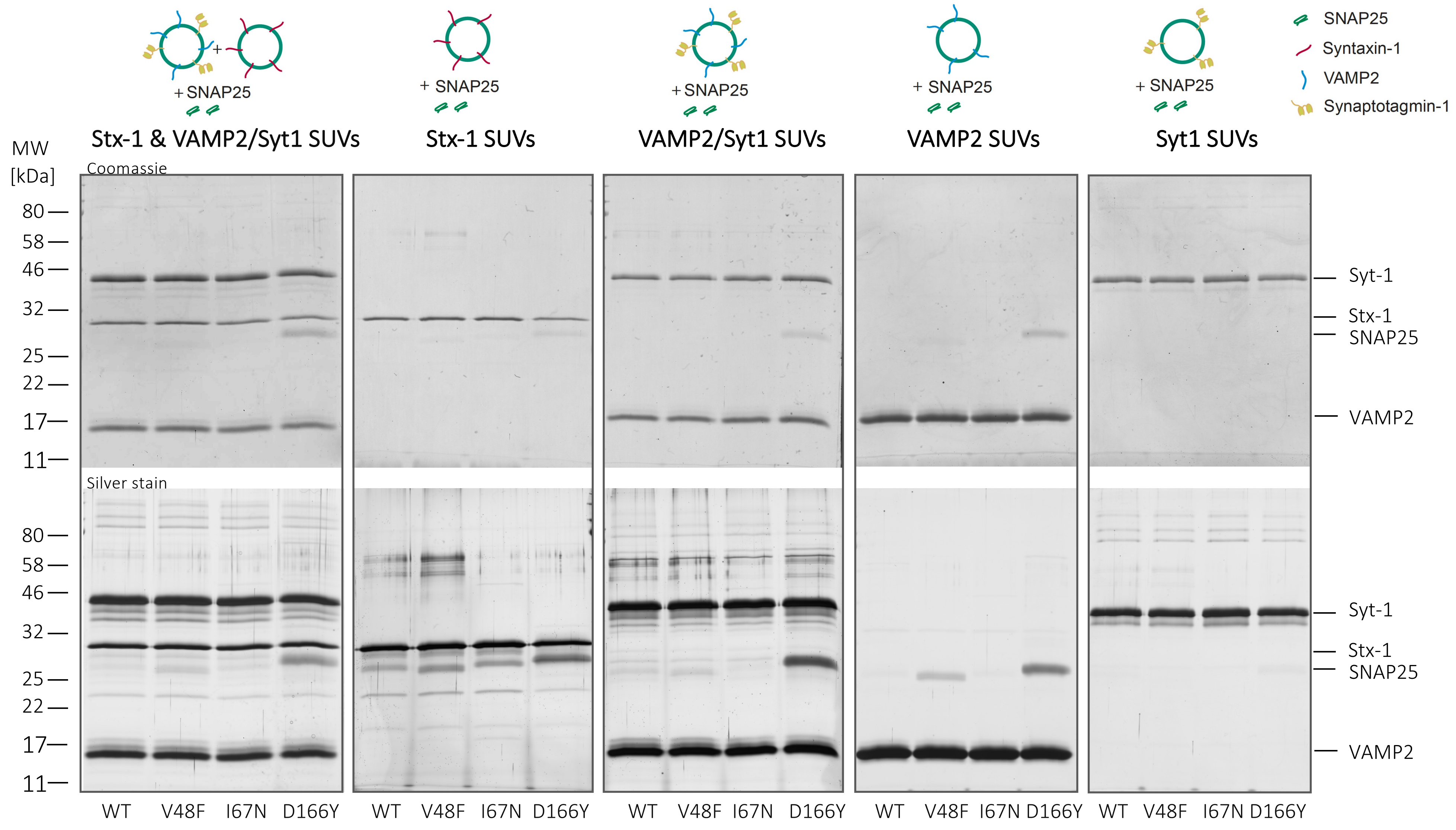

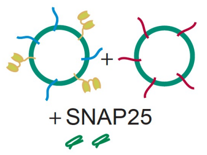

Stx-1 & VAMP2/Syt1 SUVs

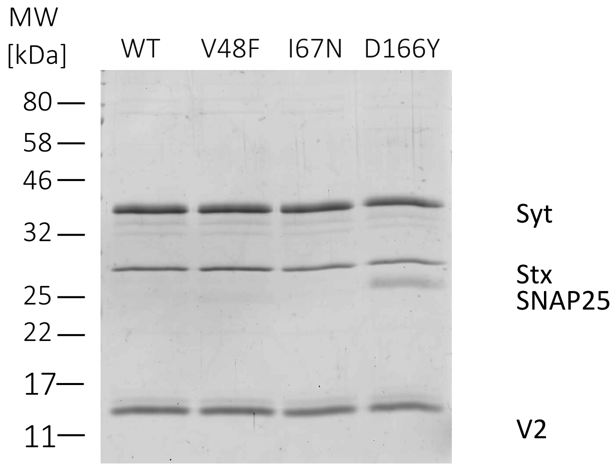

Coomassie

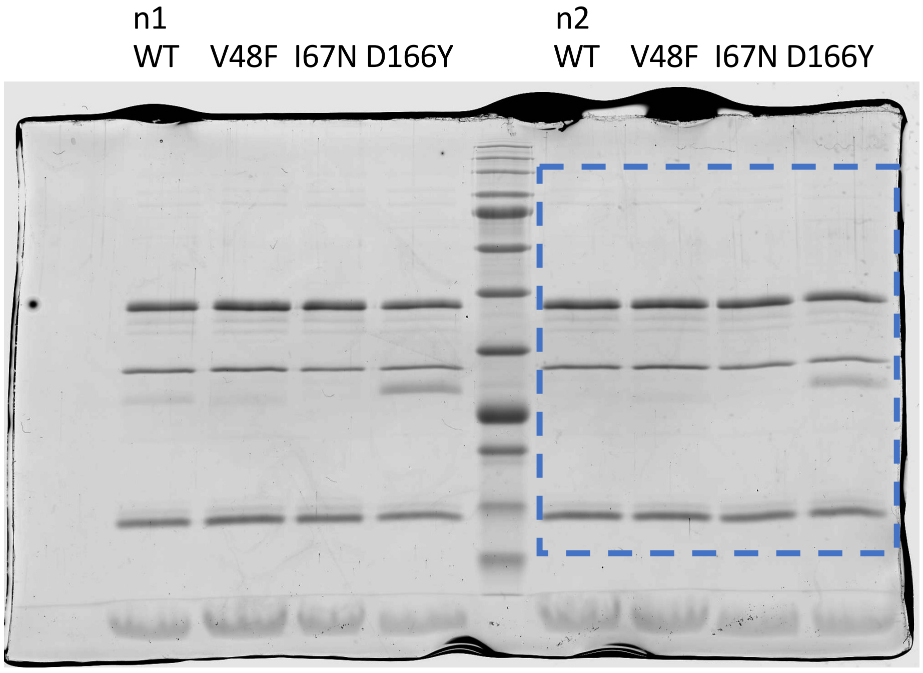

Coomassie

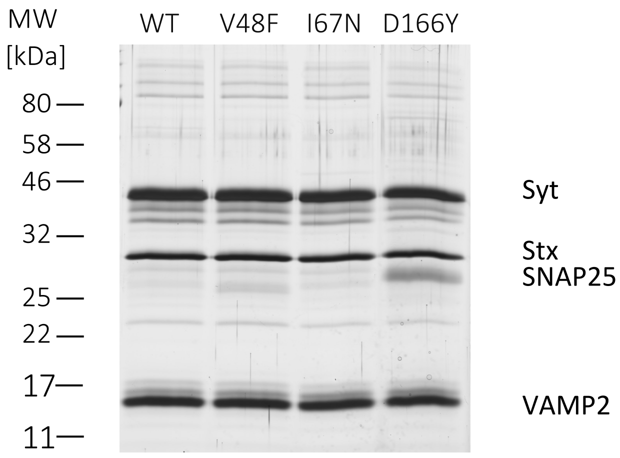

Silver stain

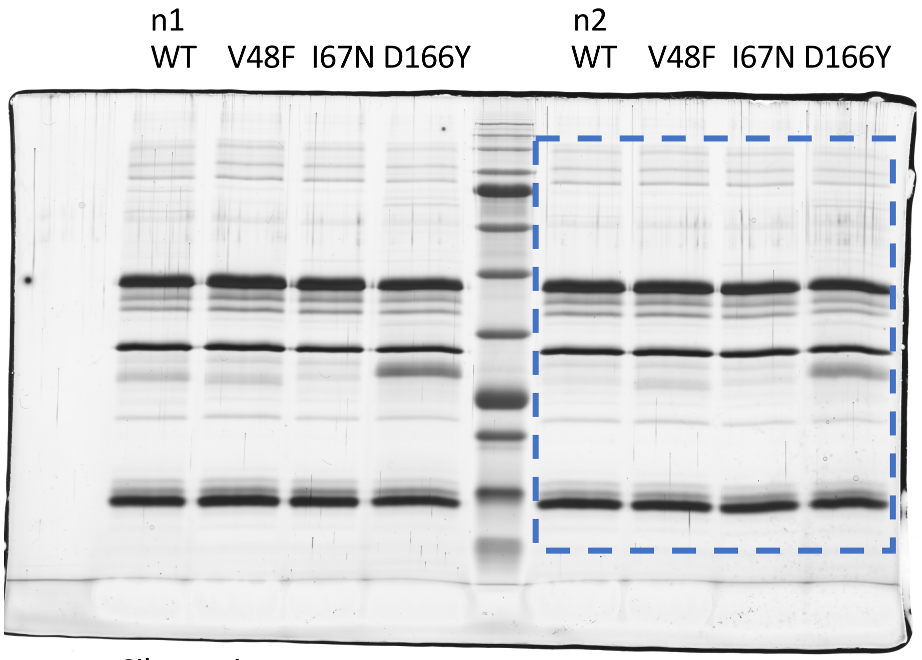

Silverstain

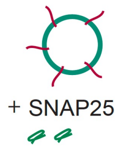

Stx-1 SUVs

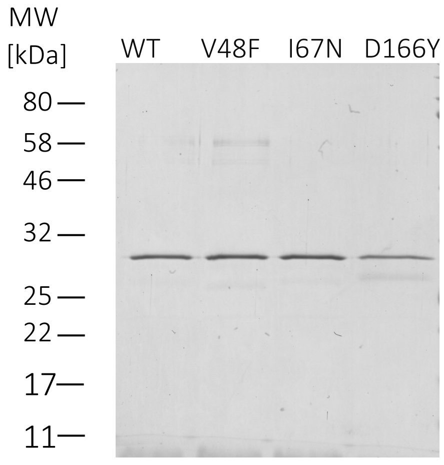

Coomassie

Stx
SNAP25

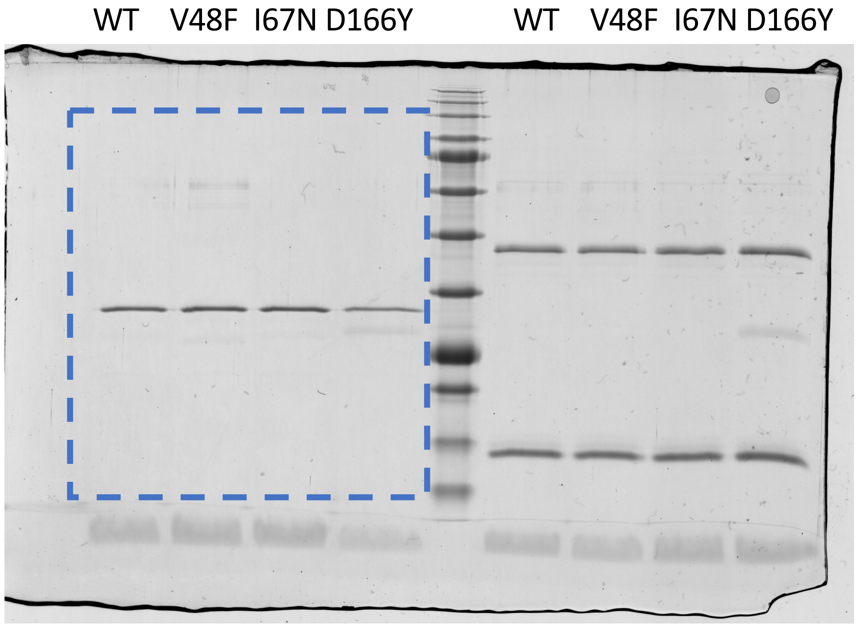

Coomassie

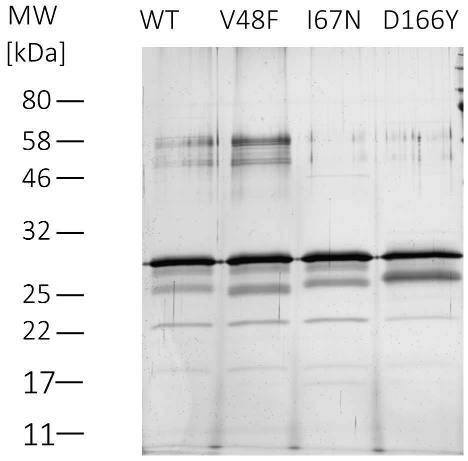

Silver stain

Stx
SNAP25

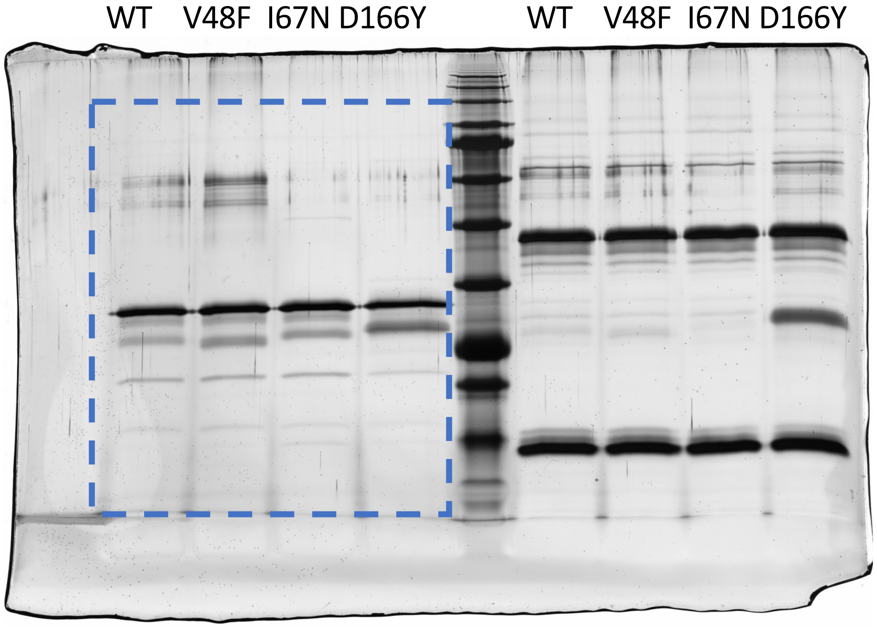

Silverstain

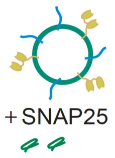

VAMP2/Syt1 SUVs

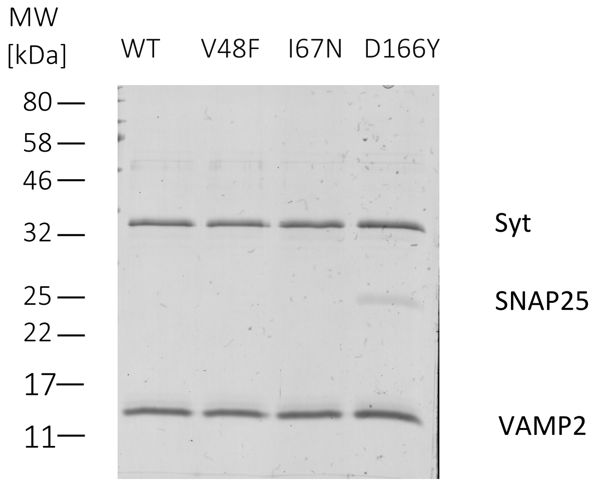

Coomassie

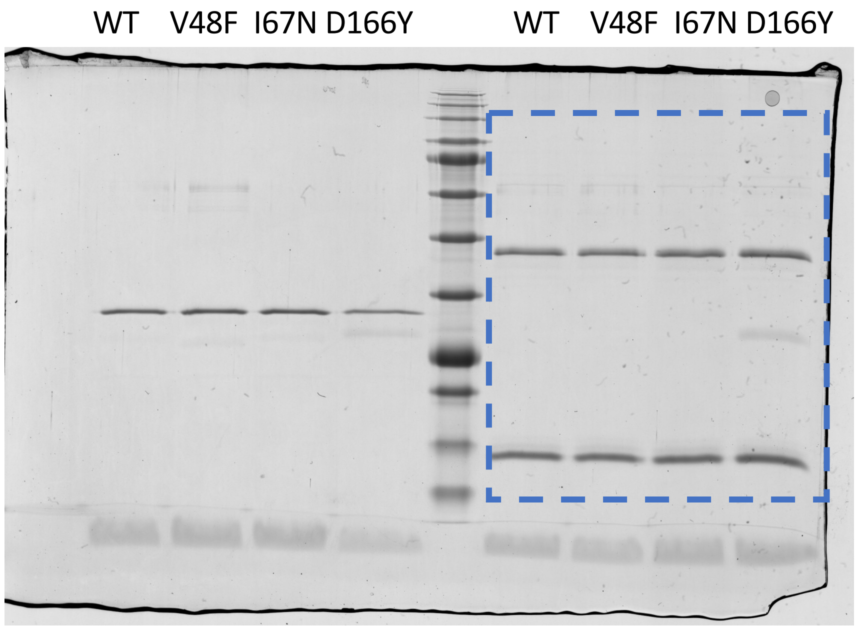

Coomassie

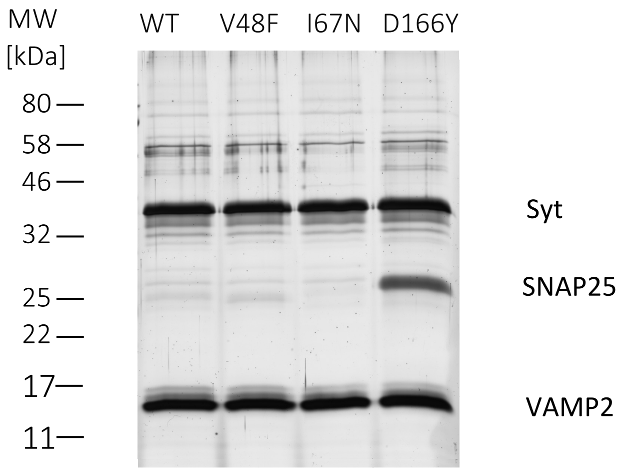

Silver stain

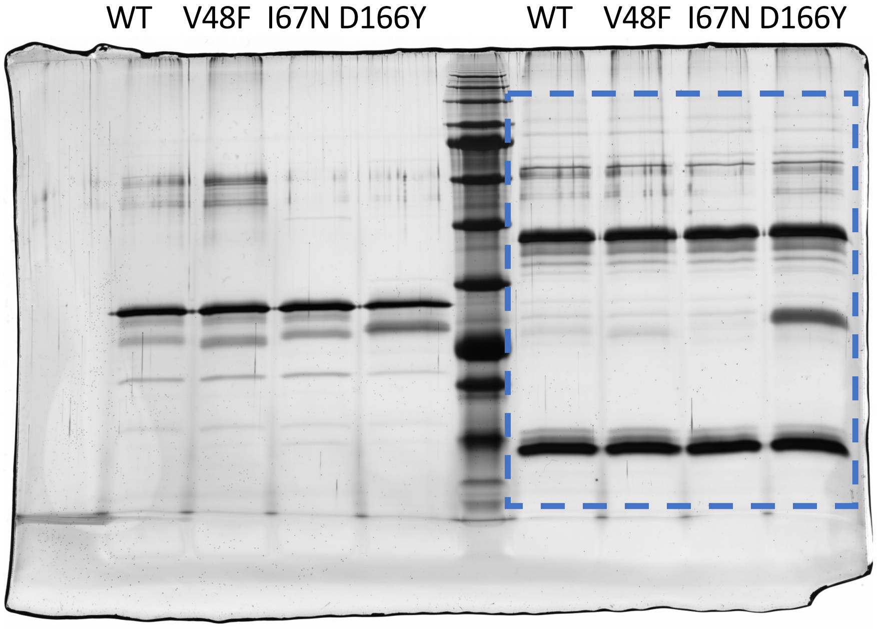

Silverstain

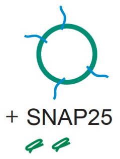

VAMP2 SUVs

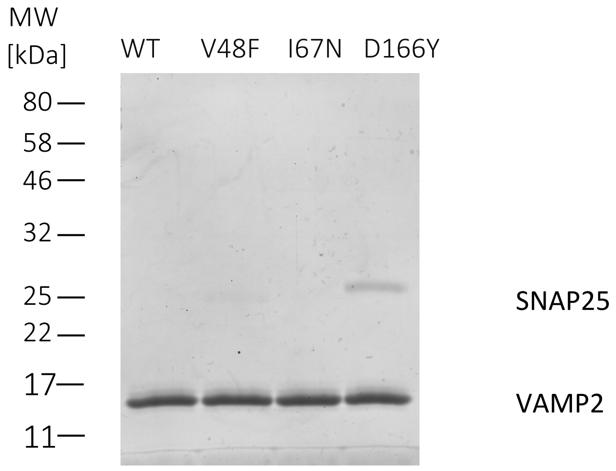

Coomassie

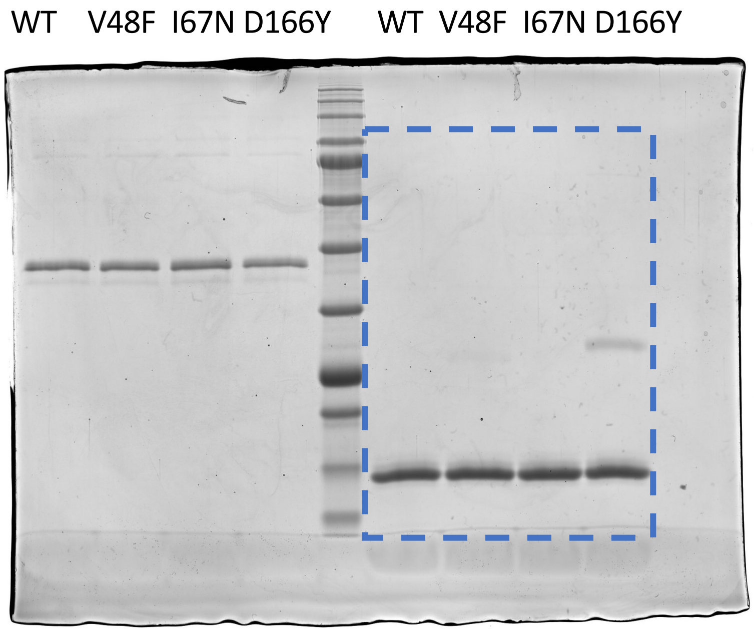

Coomassie

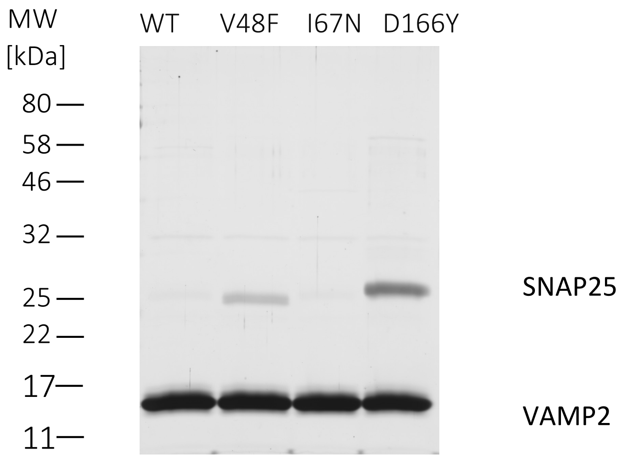

Silver stain

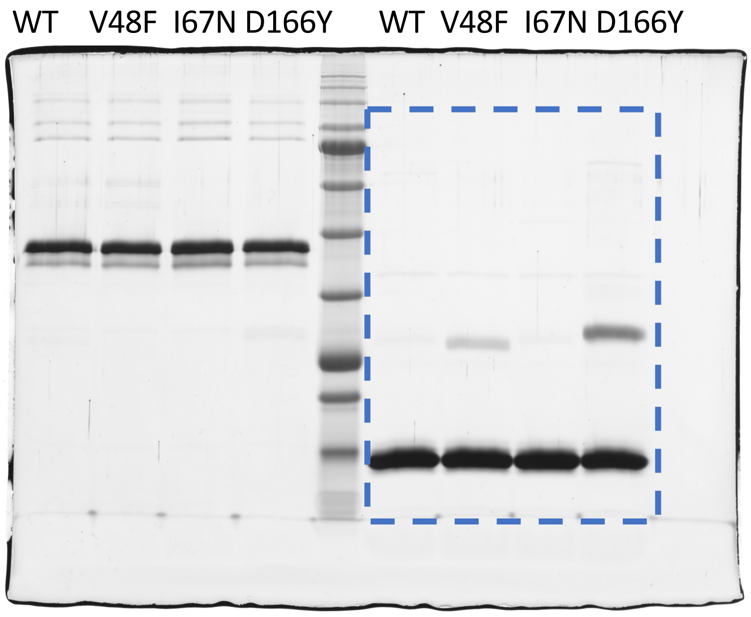

Silverstain

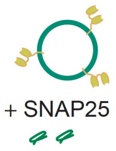

Syt1 SUVs

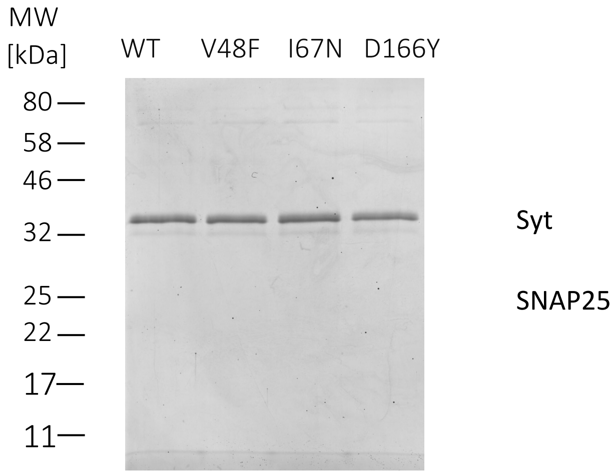

Coomassie

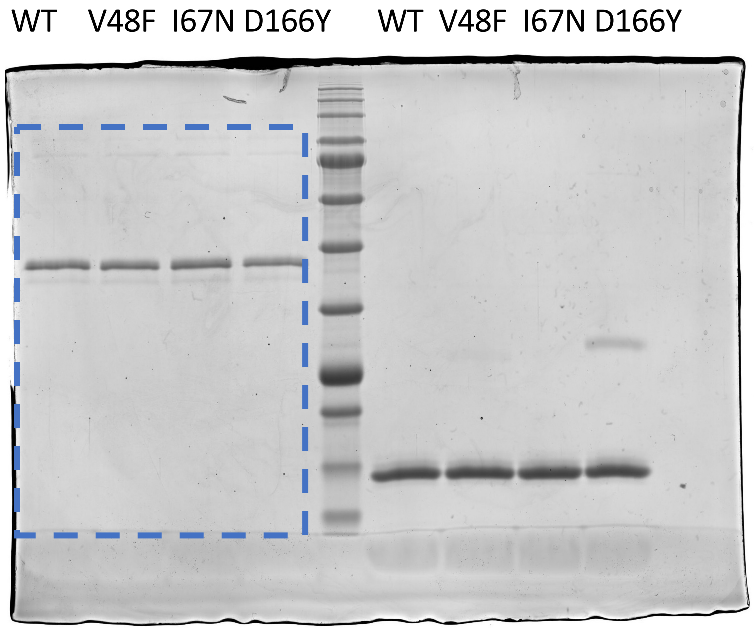

Coomassie

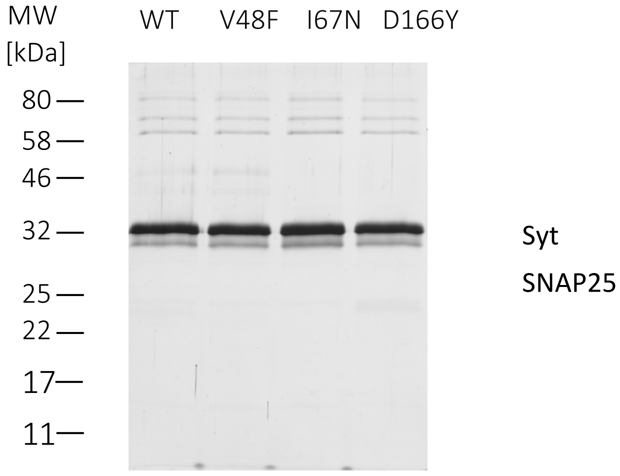

Silver stain

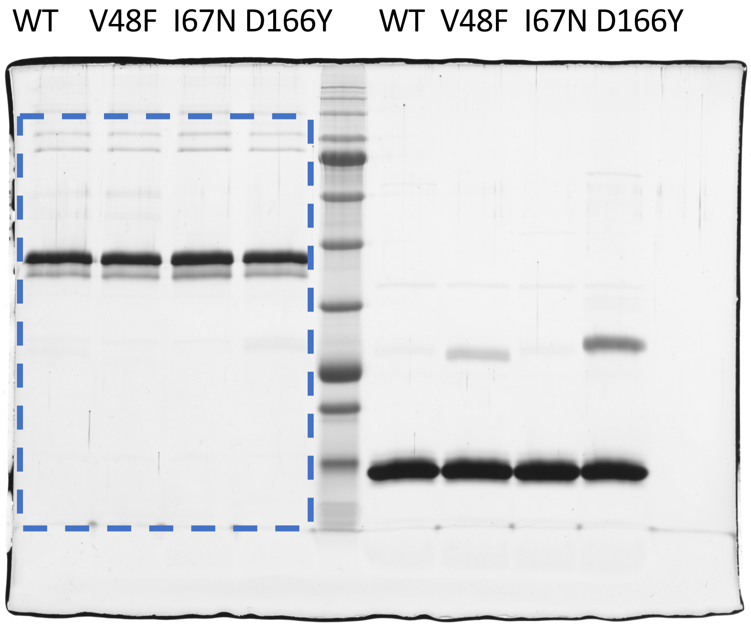

Silverstain
